# The Relationship Between Therapeutic Communication Skills and Spiritual Care Competencies of Nurses: The Example of Türkiye

**DOI:** 10.1007/s10943-025-02348-w

**Published:** 2025-06-11

**Authors:** Havva Akpinar, Fatmanur Ozcan Mircali, Nezihe Bulut Ugurlu

**Affiliations:** 1 Department of Psychiatric Nursing, Faculty of Health Sciences, Mugla Sıtkı Kocman University, Mugla, Türkiye; 2 Department of Nursing, Institute of Health Sciences, Mugla Sıtkı Kocman University, Mugla, Türkiye

**Keywords:** Spiritual care, Spirituality, Therapeutic communication, Nursing care

## Abstract

This study aims to examine the relationship between nurses' therapeutic communication skills and their spiritual care competencies. A cross-sectional, correlational research design was used. The study sample consisted of 215 nurses employed at a training and research hospital in Türkiye (n = 215). Data were collected using a descriptive data form, the Therapeutic Communication Skills Scale for Nurses, and the Spiritual Care Competence Scale. As a result of this research, it was determined that the therapeutic communication skills of the nurses were moderate, their spiritual care competence was high, and almost all of the nurses thought that it was necessary to provide spiritual care to patients and that spiritual care had an effect on the recovery of patients. A positive correlation was found between nurses' therapeutic communication skill levels and spiritual care competencies. In addition, higher levels of therapeutic communication skills were found to affect spiritual care competencies positively. In line with these results, it is thought that increasing the therapeutic communication skills of nurses will increase their spiritual care competence, and it is recommended that initiatives be carried out to increase the therapeutic communication skill levels of nurses and similar studies be carried out in different institutions.

## Introduction

Human beings have biological, social, and spiritual needs. For individuals facing chronic illnesses, crises, or life challenges, their spiritual needs often intensify along with their physical, emotional, and social needs (İsmailoğlu et al., 2019). Spiritual needs include fundamental concepts such as hope, trust, love, honesty, prayer, and worship. Addressing these needs and providing spiritual support is critical in helping patients cope with challenging situations (Ramezani et al., 2014). Nurses play a pivotal role in providing spiritual support to patients. In nursing, spirituality extends beyond religious practices and includes helping patients find meaning in life, promoting inner peace, and problem-solving, and empowering them to face illness and life challenges with resilience (Özer et al., 2019).

Spiritual care addresses the spiritual needs of individuals within the context of nursing practice. Spiritual care is a human-centered, socially oriented personal care service designed to enhance the morale and development of those needing care, promote their inner peace, and provide support in times of crisis (Karaman & Macit, 2019). Spiritual care involves being present with individuals who are experiencing crisis or emotional upheaval due to adverse life events and includes providing support, guidance, and counseling to help them find meaning in their experiences and lives (Diego-Cordero et al., 2022). Another perspective defines spiritual care as a form of personal care rooted in unconditional love that helps individuals recognize their inherent worth, which is shaped by the nurse's spiritual and cultural beliefs, emotions, and thoughts (Erişen & Sivrikaya, 2017; Wittenberg et al., 2017). Spiritual care not only enhances an individual's ability to cope with crisis but also improves their quality of life. As an integral part of nursing care, spiritual care requires the provision of personalized spiritual care, unique for each patient (Diego-Cordero et al., 2022).

Spiritual care contributes significantly to patients' healing, resilience, and overall satisfaction, positively impacting their quality of life (Huehn et al., 2019; Panczyk et al., 2021). Spiritual care provides a sense of empowerment, reduces pain and anxiety, and provides psychological comfort. In addition, holistic care, which includes spiritual care, has become a key expectation of modern healthcare services. Addressing patients' spiritual needs not only improves their well-being but also promotes engagement and positive perceptions of the nursing profession (Diego-Cordero et al., 2022).

The primary goal of nursing is to provide holistic care to patients and achieve optimal health outcomes. Holistic care encompasses physical, mental, and spiritual care, in an interconnected manner. Spirituality plays a critical role in helping individuals find hope and meaning during their most vulnerable moments. Nurses are responsible for conducting spiritual assessments, identifying spiritual distress, and providing appropriate spiritual care support to their patients (Huehn et al., 2019).

Effective spiritual care requires nurses to have strong communication skills to address the needs and desires of patients (Özcan & Akpınar, 2023; Panczyk et al., 2021). Nursing interventions that address patients' spiritual needs include respecting the patient, encouraging self-expression, active listening, using therapeutic communication techniques, and using therapeutic touch appropriately. In addition, nurses should honor patients' prayer and worship practices and involve family members in spiritual care when appropriate (Özcan & Akpınar, 2023; Wittenberg et al., 2017). Specific spiritual care practices for nurses may include respecting objects that are meaningful to patients, assisting with prayers, offering a comforting smile, playing music, answering questions, being attentive during painful procedures, reading to patients, allaying concerns, and incorporating complementary therapies such as aromatherapy, massage, and counseling (Boztilki & Ardıç, 2017; Bulut & Meral, 2019; Wittenberg et al., 2017).

Therapeutic communication fosters trust between nurses and patients, supports patient-centered care, and strengthens the bond between nurses and patients (Umeron, 2017). To understand patients' spiritual care needs and provide effective spiritual support, nurses must establish accurate and therapeutic communication with patients. It is critical for patient well-being that nurses' spiritual care competencies meet patients' expectations and are conveyed through appropriate communication manners (Çetintaş et al., 2021; Han et al., 2023). In a study by Adams et al. (2017), nurses working in intensive care units emphasized the importance of communication with patients and their families as a critical component of their nursing role, highlighting the need for communication training to effectively address barriers.

It is critical for patient well-being that nurses' spiritual care competencies meet the expectations of patients and are conveyed through appropriate communication. Given the significant role of nurses in patient care, the ability to use therapeutic communication effectively is critical to providing appropriate spiritual care. High levels of therapeutic communication skills in nurses are predicted to play a critical role in understanding and addressing patients' spiritual needs, thereby improving the level of spiritual care provided. This study aims to examine the relationship between therapeutic communication skills and spiritual care competencies of nurses.

## Research Questions

- What are the levels of nurses' therapeutic communication skills?
- What are the spiritual care competency levels of nurses?
- How do nurses' descriptive characteristics relate to their therapeutic communication skill levels and spiritual care competency levels?
- What is the relationship between nurses' therapeutic communication skills and their spiritual care competencies?

## Materials and Methods

### Research Design

This study used a cross-sectional and correlational research design.

### Participants

The study population consisted of nurses working in a training and research hospital in Türkiye (N = 401). The sample size was determined using G*Power 3.1.9.7 software (Kang, 2021). Following Cohen's (1988) recommendation for a moderate effect size (f^2^ = 0.25), a 95% confidence interval, and a 5% margin of error (Polit & Beck, 2021), the required sample size was calculated to be 190 participants.

To account for potential attrition, the sample size was increased by 10% (Akbulut, 2021) to a minimum of 209 participants. Ultimately, 215 nurses who agreed to participate and accurately completed the data collection forms were included in the study (n = 215).

### Data Collection Process

The study data were collected by the researcher through face-to-face interviews conducted at the hospital between October 1, 2023, and December 31, 2023. These interviews were scheduled during the nurses' available hours to avoid disrupting their workflow, and the questionnaires were distributed and completed within approximately 10 min.

### Data Collection Instruments

The Introductory Information Form, the Therapeutic Communication Skills Scale for Nurses, and the Spiritual Care Competence Scale were used in the study to collect data.

### Introductory Information Form

The introductory information form was developed by the researchers based on relevant literature (Dağhan et al., 2019; İsmailoğlu et al., 2019; Karaca et al., 2019). It included questions on demographic details such as age, gender, educational background, years of employment, work unit, communication, and perspectives on spiritual care.

### Therapeutic Communication Skills Scale for Nurses

The scale was developed by Karaca et al., (2019) under the name of “Therapeutic Communication Skills Scale for Nursing Students” to measure the therapeutic communication skills of nursing students. The “Therapeutic Communication Skills Scale for Nursing Students” scale was used in this study as “Therapeutic Communication Skills Scale for Nurses (TCSSN)” with the permission and approval of the responsible author who developed the scale. The TCSSN is a 7-point Likert self-rating scale (1 = "never," 4 = "sometimes," 7 = "always") with no reverse-coded items. Higher scale scores indicate greater therapeutic communication skills. The scale consists of 16 items. The lowest and highest scores on the scale are 16 and 112, respectively. The scale consists of three sub-dimensions: Non-Therapeutic Communication Skills, which reflects recognition of approaches that hinder communication; Therapeutic Communication Skills I, which represents the use of techniques that facilitate nurse-patient communication; Therapeutic Communication Skills II, which focuses on the use of communication skills that are essential for initiating and maintaining effective nurse-patient communication. In the reliability analysis of the original study with students, Cronbach's alpha values were α = 0.77 for the total scale, α = 0.82 for the non-therapeutic communication skills sub-dimension, α = 0.79 for the therapeutic communication skills I sub-dimension, and α = 0.60 for the therapeutic communication skills II sub-dimension (Karaca et al., 2019).

In this study, the reliability analysis showed higher Cronbach's alpha values with α = 0.90 for the total scale, α = 0.80 for non-therapeutic communication skills, α = 0.78 for therapeutic communication skills I, and α = 0.78 for therapeutic communication skills II. The Cronbach's alpha value for the Turkish validity and reliability study of the scale was between α = 0.78 and α = 0.90. A Cronbach's alpha value of α = 0.70 or higher indicates a reliable measurement tool (Büyüköztürk et al., 2024). Based on these Cronbach's alpha values, the scale is considered a reliable instrument for assessing nurses' therapeutic communication skills.

### Spiritual Care Competence Scale

The Spiritual Care Competence Scale (SCCS), originally developed by Leeuwen et al. (2009), was validated and adapted to Turkish by Dağhan et al. (2019). This scale consists of 27 items and measures three sub-dimensions of spiritual care: assessment and implementation of spiritual care, professionalism and development of spiritual care, and personal support, including patient counseling, guidance, and communication. It's a five-point Likert scale. The lowest and highest scores on the scale are 27 and 135 respectively. Higher scores indicate greater nursing competence regarding spiritual care. In the Turkish adaptation study, Cronbach's alpha values showed excellent internal consistency with α = 0.97 for the total scale, α = 0.94 for the spiritual care assessment and implementation sub-dimension, α = 0.96 for professionalism and patient counseling, and α = 0.96 for patient attitudes toward spirituality and communication (Dağhan et al., 2019). In this study, the SCCS also demonstrated strong reliability, with a Cronbach's alpha of α = 0.91 for the total scale, α = 0.93 for the assessment and implementation sub-dimension, α = 0.84 for professionalism and patient counseling, and α = 0.95 for patient attitudes toward spirituality and communication sub-dimension.

### Data Analysis

Study data were analyzed using IBM SPSS Statistics 25.0 (SPSS Inc., Chicago, IL, USA) software. Descriptive characteristics of the participating nurses were assessed using frequency and percentage analyses, while mean and standard deviation statistics were used to evaluate scale data. The normality of the research variables was assessed using kurtosis and skewness values, which confirmed that the variables were normally distributed. The variables were found to be normally distributed. The t-test, one-way analysis of variance (ANOVA), and post hoc analysis (Tukey, LSD) were used to examine the differences in scale scores according to the descriptive characteristics of the nurses. Parametric methods were used throughout the data analysis. Relationships between scale dimensions were examined using Pearson correlation and linear regression analyses. Cronbach's alpha value, 95% confidence interval, and a p-value of less than 0.05 for statistical significance were used for reliability analysis (Büyüköztürk et al., 2024; Schober et al., 2018).

### Ethical Considerations

This study was approved by the Ethics Committee of a state university (Medical and Health Sciences Ethics Committee-2 [Sports, Health], Date: 14.05.2023, Decision No: 230055-70). Institutional approval was also obtained from the institution where the research was conducted, and permission to use the measurement scales was obtained from the authors, who adapted these scales into Turkish and verified their validity and reliability. Participants were recruited voluntarily. They were fully informed about the study through an informed consent form, and only those who consented were included in the study. Confidentiality of all data collected was strictly maintained. Participants were explicitly informed that participation in the study was completely voluntary, that no identifying names or signatures were required, that they could withdraw at any time, and that all information would remain confidential. Equal treatment of all participants was assured. The study adhered to the ethical standards of the World Medical Association's Declaration of Helsinki, particularly the Ethical Principles for Medical Research Involving Human Subjects 2013/64, in line with the principles of publication ethics.

## Results

Analysis of the descriptive characteristics of the participating nurses revealed that 34% were 38 years of age or older, 80.5% were female, and 43.7% had more than 11 years of professional experience. The majority, 51.2%, worked in intensive care or emergency services, 67.4% were familiar with the concepts of therapeutic and nontherapeutic communication, 85.1% were familiar with the concept of spiritual care, 91.6% believed that providing spiritual care was necessary, and 95.3% believed that spiritual care positively influenced patient recovery (Table 1).

**Table 1:** Distribution of nurses according to descriptive characteristics

Descriptive characteristics		n	%
Age X̄ = 34.10 ± 8.80 (Min = 20, Max = 55)	18–27	71	33.0
	38 and above	73	34.0
Gender	Female	173	80.5
	Male	42	19.5
Working time X̄ = 12.08 ± 9.96 (Min = 1, Max = 37)	1–5 years	85	39.5
	6–10 years	36	16.7
	11 years and above	94	43.7
Work Unit	Internal/surgical units	105	48.8
	Intensive care/emergency service	110	51.2
Do you know the concepts of therapeutic/non-therapeutic communication?	Yes	145	67.4
	No	70	32.6
Do you know the concept of spiritual care?	Yes	183	85.1
	No	32	14.9
Is it necessary to provide spiritual care to patients?	Yes	197	91.6
	No	18	8.4
Does spiritual care affect the recovery of patients?	Yes	205	95.3
	No	10	4.7
TOTAL		215	100

According to the scores of the nurses on the scales, the mean total score for the TCSSN was 72.28 ± 15.80. The sub-dimension scores were 30.13 ± 8.82 for Non-Therapeutic Communication Skills, 28.07 ± 6.67 for Therapeutic Communication Skills I, and 15.23 ± 3.44 for Therapeutic Communication Skills II. For the SCCS, the mean total score was 99.75 ± 16.88. The sub-dimension scores were 21.08 ± 4.85 for spiritual care assessment and implementation, 53.36 ± 10.69 for professionalism in spiritual care and patient counseling, and 25.31 ± 4.84 for patient attitudes toward spirituality and communication (Table 2).

**Table 2:** Distribution of the mean scores on the TCSSN and SCCS and their sub-dimensions (n = 215)

Scale score averages	X̄ ± SD
TCSSN total score *Sub-dimensions* Non-therapeutic communication skills Therapeutic communication skills I Therapeutic communication skills II	72.28 ± 15.80 30.13 ± 8.82 28.07 ± 6.67 15.23 ± 3.44
SCCS total score *Sub-Dimensions* Assessment and implementation of spiritual care Professionalism in spiritual care and patient counseling Patient's attitude towards spirituality and communication	99.75 ± 16.88 21.08 ± 4.85 53.36 ± 10.69 25.31 ± 4.84

*TCSSN* therapeutic communication skills scale for nurses; *SCCS* spiritual care competence scale

Table 3 presents the findings related to differences between several descriptive characteristics of the nurses and scale total scores. Accordingly, nurses aged 38 years and older (78.00 ± 16.95) scored higher on the TCSSN than those aged 18–27 years (70.68 ± 12.97). Similarly, those with more than 11 years of working experience (78.37 ± 16.26) scored higher than those with 1–5 years of experience (71.44 ± 13.65). Nurses working in medical or surgical units (78.02 ± 16.50), those familiar with therapeutic and non-therapeutic communication concepts (76.52 ± 16.57), and those who considered spiritual care essential (77.04 ± 15.66) also had higher TCSSN scores. Nurses working in medical or surgical units (78.02 ± 16.50), those familiar with therapeutic and non-therapeutic communication concepts (76.52 ± 16.57), and those who considered spiritual care essential (77.04 ± 15.66) also had higher TCSSN scores. Higher SCCS scores were observed among nurses familiar with therapeutic and non-therapeutic communication concepts (101.94 ± 16.28) and spiritual care (101.15 ± 16.25), as well as those who considered spiritual care necessary (101.63 ± 15.28) and believed it affected patient recovery (100.76 ± 15.95). These results were statistically significant (*p* < 0.05, Table 3).

**Table 3:** Difference between nurses' descriptive characteristics and total mean scores on the TCSSN and SCCS (n = 215)

Descriptive characteristics	TCSSN total score X̄ ± SD Test and *p*	SCCS total score X̄ ± SD Test and *p*
*Age*		
18–27^1^ 28–37^2^ 38 and above^3^ Post-hoc =	70.68 ± 12.97 74.06 ± 16.51 78.00 ± 16.95 F = 3.982 *p* = .020 3 > 1	100.65 ± 18.30 97.62 ± 16.50 100.95 ± 15.81 F = 0.848 *p* = .430
*Working time*		
1–5 years^1^ 6–10 years^2^ 11 years and above^3^ Post-hoc =	71.44 ± 13.65 70.31 ± 17.24 78.37 ± 16.26 F = 5.925 *p* = .003 3 > 1	99.884 ± 18.18 94.64 ± 17.31 101.59 ± 15.19 F = 2.234 *p* = .110
*Work unit*		
Internal/surgical units Intensive care/emergency service	78.02 ± 16.50 70.71 ± 14.30 t = − 3.476 *p* = .001	100.04 ± 15.07 99.47 ± 18.51 t = 0.245 *p* = .807
*Do you know the concepts of therapeutic/non-therapeutic communication?*		
Yes No	76.52 ± 16.57 69.64 ± 13.02 t = 3.045 *p* = .003	101.94 ± 16.28 95.21 ± 17.29 t = 2.780 *p* = .002
*Do you know the concept of spiritual care?*		
Yes No	74.38 ± 15.88 73.72 ± 15.62 t = 0.217 *p* = .829	101.15 ± 16.25 91.75 ± 18.39 t = 2.958 *p* = .003
*Is it necessary to provide spiritual care to patients?*		
Yes No	77.04 ± 15.66 69.63 ± 15.04 t = 3.005 *p* = .001	101.63 ± 15.28 79.17 ± 20.08 t = 5.803 *p* = .000
*Does spiritual care affect the recovery of patients?*		
Yes No	74.31 ± 15.78 73.70 ± 17.31 t = 0.118 *p* = .906	100.76 ± 15.95 79.00 ± 22.52 t = 4.128 *p* = .000

*TCSSN*: therapeutic communication skills scale for nurses, *SCCS* spiritual care competence scale

The correlation between the mean scores of the TCSSN and the SCCS was analyzed using Pearson correlation analysis, and the results are presented in Table 4. The analysis revealed a weak positive correlation between the total TCSSN score and the total SCCS score (r = 0.270, *p* = 0.000). Similarly, weak positive correlations were observed between the total TCSSN score and the SCCS sub-dimensions of professionalism in spiritual care and patient counseling (r = 0.294, *p* = 0.000) and patient attitudes toward spirituality and communication (r = 0.250, *p* = 0.000). A weak positive relationship was also found between the SCCS total score and the TCSSN sub-dimensions Therapeutic Communication Skills I (r = 0.290, *p* = 0.000) and Therapeutic Communication Skills II (r = 0.291, *p* = 0.000). In addition, weak positive correlations were found between the SCCS Spiritual Care and Patient Counseling sub-dimensions and the TCSSN Therapeutic Communication Skills I (r = 0.306, *p* = 0.000) and Therapeutic Communication Skills II (r = 0.284, *p* = 0.000) sub-dimensions. The SCCS sub-dimension patient's attitude toward spirituality and communication also showed weak positive correlations with the TCSSN sub-dimensions Therapeutic Communication Skills I (r = 0.237, *p* = 0.000) and Therapeutic Communication Skills II (r = 0.327, *p* = 0.000) (*p* < 0.05, Table 4).

**Table 4:** The relationship between scores of nurses on the TCSSN and SCCS (n = 215)

TCSSN		SCCS			
		Spiritual care competence scale total score	Assessment and implementation of spiritual care sub-dimension	Professionalism in spiritual care and patient counseling sub-dimension	Patient's attitude toward spirituality and communication sub-dimension
Therapeutic Communication Skills Scale for Nurses Total Score	Cor	.270^*^	.040	.294^*^	.250^*^
	p	.000	.558	.000	.000
Non-therapeutic Communication Skills Sub-Dimension	Cor	.117	-.061	.157	.122
	p	.088	.374	.122	.075
Therapeutic Communication Skills I Sub-Dimension	Cor	.290^*^	.097	.306^*^	.237^*^
	p	.000	.155	.000	.000
Therapeutic Communication Skills II Sub-Dimension	Cor	.291^*^	.061	.284^*^	.327^*^
	p	.000	.375	.000	.000

*TCSSN* therapeutic communication skills scale for nurses, *SCCS* spiritual care competence scale

*p < .05, Pearson Correlation Analysis

In addition, regression analysis was performed to determine the cause and effect relationship between therapeutic communication skills and spiritual care competencies of nurses. The regression analysis performed to determine the cause and effect relationship between the nurses' TCSSN total score average and SCCS total score average was found to be significant (F = 16.699, *p* = 0.000). When the regression analysis was examined, it was determined that the equation between TCSSN and SCCS was; SCCS = 78.365 + 0.288 × TCSSN and that when the TCSSN value increased by 1 unit, the SCCS value increased by 0.288 units. Table 5).

**Table 5:** The effect of therapeutic communication skills on nurses on their spiritual care competencies with regression analysis (n = 215)

Dependent variable	Independent Variable	Non-standardized Coefficients	β	t	*p*	R^2^	Adjusted R^2^	F
SCCS	Constant	78.365		14.649	.000*	.073	.068	16.699
	TCSSN	.288	.270	4.086	.000*			

*SCCS* spiritual care competence scale, *TCSSN* therapeutic communication skills scale for nurses

*p < .05, R = Regression coefficient

## Discussion

This study, which aimed to explore the relationship between nurses' therapeutic communication skills and their spiritual care competencies, revealed a positive correlation between them. It was found that nurses with higher levels of therapeutic communication skills demonstrated stronger spiritual care competencies. In addition, nurses who were familiar with the concepts of therapeutic and non-therapeutic communication demonstrated higher spiritual care competencies. The results also showed that nurses who understood the concepts of therapeutic and non-therapeutic communication and believed in the need to provide spiritual care to patients had better therapeutic communication skills. To improve nurses' spiritual care competencies, it is important to foster advanced therapeutic communication skills, adopt a patient-centered care approach, and develop an understanding of the complex nature of spirituality while raising awareness about this topic (Gökçe et al., 2021; Irmak, 2018). When providing spiritual care, nurses should have strong therapeutic communication skills, build trusting relationships with patients, and motivate patients in their treatment journey (Güz & Demir Yaleze, 2019; Köktürk Dalcalı, 2019).

Nurses need to empathize with patients, actively listen, and use therapeutic communication techniques to assess patients' spiritual needs and alleviate their suffering (Özcan & Akpınar, 2023). A meta-analysis by Diego-Cordero (2022) highlighted that spiritual care provided by nurses positively affects patients' mental, physical, and spiritual health, reduces their suffering, and improves their quality of life. In addition, the use of therapeutic communication in this process was shown to significantly improve the effectiveness of spiritual care provided. However, a study by Huehn et al. (2019) found that nurses often felt that their lack of knowledge about spirituality and spiritual care prevented them from adequately addressing patients' spiritual needs. Similarly, a study by Güner and Akyüz (2023) demonstrated a positive relationship between nurses' levels of spirituality, patient care behaviors, and overall life satisfaction.

Studies of nursing students have also yielded a positive relationship between nursing students' perceptions of spiritual care and their levels of spiritual well-being and empathy (Wang et al., 2022), and a positive correlation between students' attitudes toward spiritual care and their communication skills (Panczyk et al., 2021). In a study similar to ours, Özcan and Akpınar (2023) observed a negative relationship between students' non-therapeutic communication skills and their spiritual care competencies, while therapeutic communication skills were positively related to spiritual care competencies. The findings of the studies and related literature are consistent with our findings. Based on these findings, it is evident that establishing therapeutic communication is critical for nurses to provide effective spiritual care to patients.

Our research showed that nurses had a high level of spiritual care competence. Nearly all participants believed that providing spiritual care to patients was important and that it positively influenced patient recovery. In addition, nurses who held this belief demonstrated higher spiritual care competencies. Similarly, Dündar and Aslan (2022) found that nurses' spiritual care competencies were above average and that nurses with spiritual care knowledge were more likely to engage in spiritual care practices. Likewise, Vogel and Schep-Akkerman (2018) found a direct relationship between nurses' spiritual care practices and their knowledge of spiritual care. Additionally, a study of psychiatric nurses by Han et al. (2023) found that positive attitudes toward spiritual care, along with relevant training and experience, significantly increased nurses' spiritual care practices. These findings are consistent with our results and highlight the critical importance of improving nurses' knowledge of spiritual care to ensure the provision of quality spiritual care to patients.

### Limitations of the Study

The scope of the study is considered a limitation since it was conducted exclusively with nurses from a single institution; as a result, the study findings are limited to the nurses who participated and the data collection instruments used in the research.

## Conclusions and Recommendations

This study revealed that nurses generally demonstrated moderate therapeutic communication skills and high spiritual care competencies. Almost all participants recognized the importance of providing spiritual care to patients and acknowledged its positive impact on patient recovery. It was observed that older nurses, who had more work experience, were familiar with the concepts of therapeutic and non-therapeutic communication, and believed in the need for spiritual care had higher levels of therapeutic communication skills. Similarly, nurses who understood the concepts of therapeutic/non-therapeutic communication and spiritual care acknowledged the need for spiritual care and recognized its role in patient recovery, and demonstrated higher levels of spiritual care competence. In addition, the most notable finding of the study was the positive correlation between nurses' therapeutic communication skills and their spiritual care competencies, indicating that improved therapeutic communication skills positively influenced spiritual care competencies. Based on these findings, it is concluded that improving nurses' therapeutic communication skills can significantly enhance their spiritual care competencies. Consequently, it is recommended that targeted interventions be implemented to develop nurses' communication skills and that similar research be conducted in different healthcare settings.

## Data Availability

The dataset analyzed in this study is not publicly available due to the subjects’ permission to use it only for data analysis and not for any other use, but it is available from the corresponding author upon a reasonable request.
